# Congenital Cytomegalovirus in a Resource-Limited Setting: A Case Report

**DOI:** 10.7759/cureus.62844

**Published:** 2024-06-21

**Authors:** Philip J Bert, Carlos A Narvaez Gaitan, Vilma E Vasquez Vado

**Affiliations:** 1 Neonatology, Centro Medico-Quirúrgico Santa Fe, Matagalpa, NIC; 2 Neonatology, Hospital Escuela Cesar Amador Molina, Matagalpa, NIC; 3 Pediatrics, Hospital Escuela Cesar Amador Molina, Matagalpa, NIC; 4 Pediatric Critical Care, Hospital Escuela Cesar Amador Molina, Matagalpa, NIC

**Keywords:** neonatal cholestasis, perinatal infection, pediatrics & neonatology, periventricular calcifications, high risk neonates, blueberry muffin syndrome, colpocephaly, low resource setting, congenital cytomegalovirus

## Abstract

Diagnosing congenital cytomegalovirus (CMV) infection in neonates, particularly in developing countries with limited resources, can be challenging. This case report and literature review highlights the clinical presentation, diagnostic challenges, and management strategies associated with congenital CMV infection in a limited-resource setting.

A female neonate born at 37 weeks and weighing 1760 grams presented with jaundice, petechial rash, and ventriculomegaly detected on prenatal ultrasound. Diagnostic workup revealed splenomegaly, thrombocytopenia, and elevated bilirubin levels, prompting suspicion of CMV infection. Serological testing confirmed CMV antibodies in the neonate, indicating severe symptomatic primary congenital infection.

Imaging studies demonstrated colpocephaly with periventricular calcifications, consistent with CMV-related neurological abnormalities. Treatment with oral valganciclovir resulted in clinical improvement without adverse effects. However, follow-up was hindered by the mother's non-compliance.

This case underscores the importance of considering CMV in the differential diagnosis of neonatal jaundice and neurological abnormalities. Despite its prevalence and clinical impact, there is no consensus on universal screening during pregnancy. Strengthening preventative measures and increasing awareness are crucial steps in addressing congenital CMV infection's public health implications.

## Introduction

Cytomegalovirus (CMV) is the most common congenital infection globally and the main infectious cause of sensorineural deafness and neurodevelopmental disorders [1]. It is also the most frequent infectious cause of congenital anomalies in developing fetuses and newborns [1]. CMV presents a significant public health issue, particularly in developing countries that often lack the resources for effective diagnosis and treatment.

CMV has a worldwide prevalence of 0.3% to 0.7% of all live births [1] and can be found in one in four women of childbearing age [2]. However, it often occurs asymptomatically, complicating timely detection. The lack of universal screening in most countries exacerbates this issue. Routine CMV screening during pregnancy is currently not recommended due to a limited understanding of the disease processes and the absence of validated early interventions that can alter the disease course [3].

This case report aims to increase awareness of congenital CMV infection, especially in clinical settings in resource-limited countries. With high diagnostic suspicion and a better understanding of this infection, we can detect and treat CMV more effectively. This approach can help prevent future auditory and cognitive sequelae by working closely with a multidisciplinary team of specialists for the care of patients affected by congenital infection.

## Case presentation

The patient is a female neonate born in a secondary care hospital in mountainous regions of Matagalpa, Nicaragua. She was born at term via spontaneous vaginal delivery at a gestational age of 37 weeks, with an estimated birth weight of 1760 grams. The mother, a primigravida nulliparous woman, was diagnosed with gestational diabetes at 24 weeks and mild anemia. Her blood type was A+, with negative results for HIV, rapid plasma reagin (RPR), and toxoplasmosis. Prenatal testing for cytomegalovirus was not performed, and no symptomatic infections were reported before or during pregnancy. A prenatal obstetric ultrasound at 34 weeks reported ventriculomegaly.

**Figure 1:**
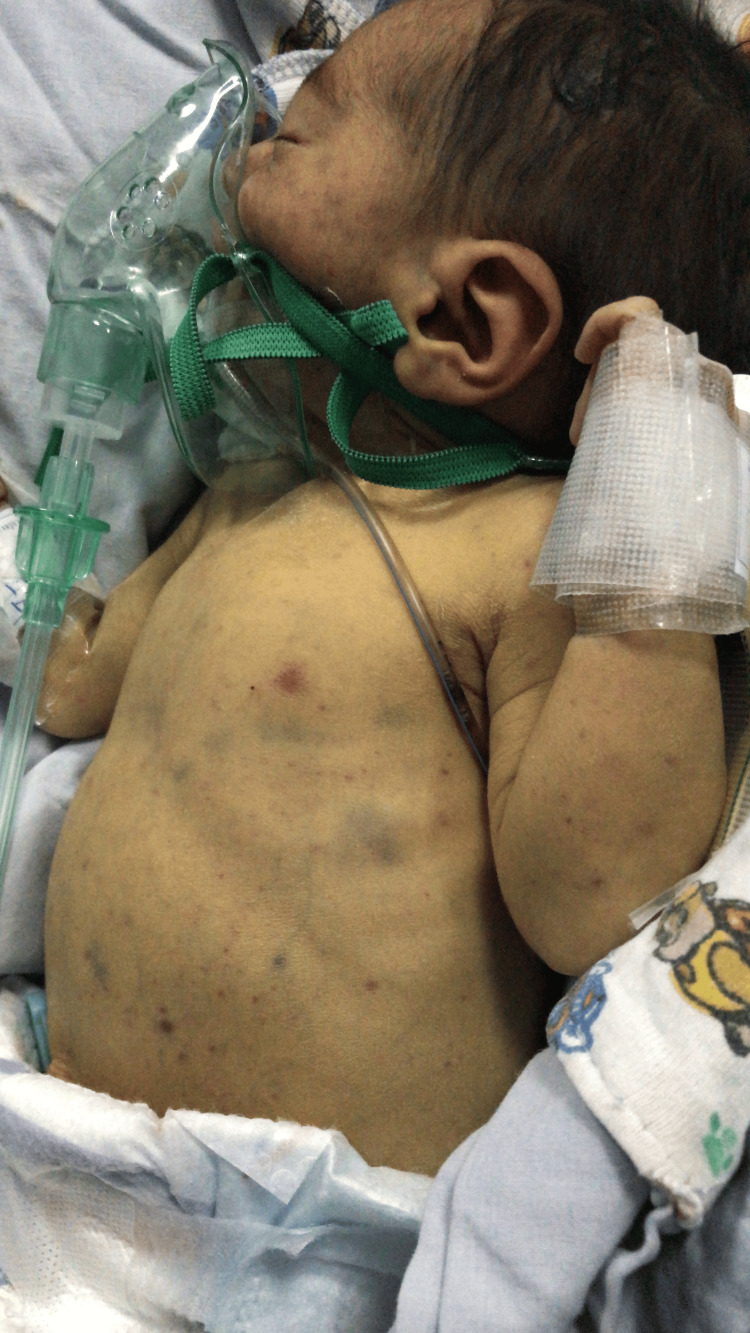
Widespread blue and purple maculopapular lesions typically seen in blueberry muffin baby syndrome

The neonate's Apgar scores were 8 and 9 at the first minute and five minutes, respectively. Physical examination revealed no apparent physical malformations, no hepatosplenomegaly, afebrile status, normal blood glucose, height of 43 cm, head circumference of 30 cm, and symmetrical intrauterine growth restriction. She was admitted to the neonatal ward for surveillance due to the risk of hypoglycemia and to perform additional studies due to the ventriculomegaly noted on the prenatal ultrasound. During her second day of life, she developed splenomegaly, jaundice, a petechial rash, and widespread maculopapular lesions (Figure 1) prompting complete blood studies, a metabolic panel, a blood culture, and imaging tests.

CBC performed upon admission showed hemoglobin (Hb) of 15.8 g/dL, hematocrit (Hct) of 44.6%, blood type O+, platelets of 49,000/mL, total leukocytes of 8140/μL, lymphocytes at 45.8%, and a C-reactive protein level of 12 mg/dL. Bilirubin levels consistently increased in the first days of life peaking on the fifth day with a total bilirubin was 19.42 mg/dL, with indirect bilirubin at 13.84 mg/dL and direct bilirubin at 5.58 mg/dL. Concern over possible sepsis led to a blood culture and empirical antibiotic coverage. Phototherapy was started with adequate clinical response.

**Figure 2:**
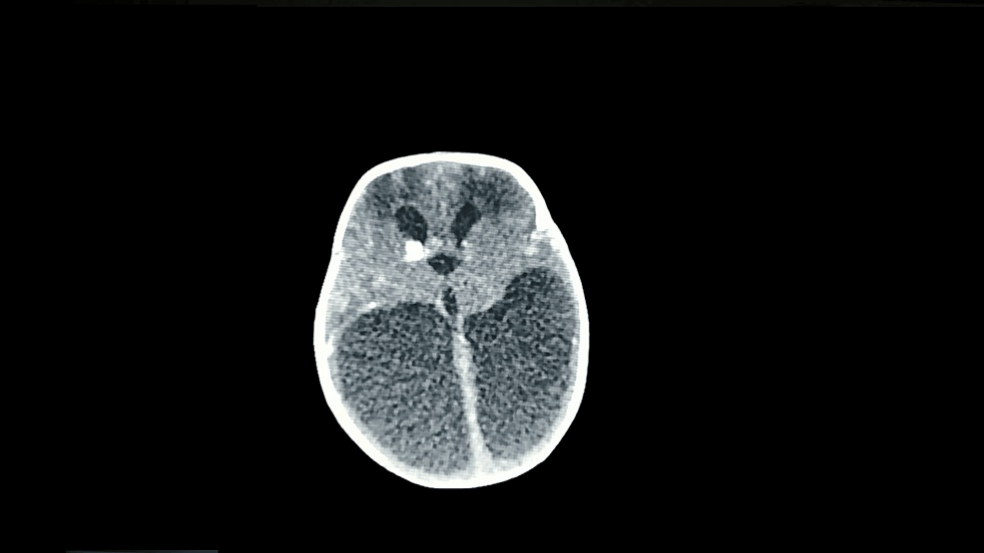
Head CT scan showing colpocephaly with multiple periventricular calcifications

A head CT scan found colpocephaly with periventricular calcifications and reduced brain parenchyma (Figure 2).

The blood culture was negative, leading to the cessation of antibiotic therapy. A blood sample for serological detection of cytomegalovirus showed immunoglobulin M (IgM) antibodies against CMV at 0.37 IU and IgG antibodies against CMV at 183.9 IU. CMV-PCR testing was not available. Given the clinical laboratory results and brain imaging findings, severe symptomatic primary congenital infection was suspected. Antiviral treatment was requested, and after obtaining consent from the baby's mother, oral valganciclovir was started at a dose of 16 mg/kg every 12 hours for 10 days. The patient showed satisfactory clinical and paraclinical improvement without reported adverse reactions. She was discharged after a 23-day NICU stay with a prescription for antiviral treatment for six months, followed by outpatient consultation and treatment follow-up through primary care. However, the baby's mother did not attend subsequent follow-up consultations, preventing further ophthalmological and auditory evaluations.

## Discussion

This case represents a typical presentation of cytomegalovirus, characterized by clinical, laboratory, and imaging findings. Common clinical signs include hepatosplenomegaly (60%), microcephaly (53%), jaundice (67%), petechiae (76%), and at least one neurological anomaly (68%). Laboratory abnormalities include increased transaminases (83%), thrombocytopenia (77%), direct hyperbilirubinemia (69%), hemolysis (51%), and hyperproteinorrachia (46%) [4]. In this case, the neonate presented splenomegaly, and jaundice, with a notable increase in direct bilirubin probably due to cholestasis caused by CMV invasion of the intrahepatic bile duct epithelium [5].

Common causes of neonatal cholestatic jaundice include biliary atresia, idiopathic hepatitis, sepsis, alpha-1 antitrypsin deficiency, and galactosemia [6]. These should be considered before diagnosing CMV infection. Many cases of perinatal CMV infection go undiagnosed in developing countries due to limited disease knowledge and diagnostic equipment shortages [6]. In this case, the jaundice was primarily associated with CMV infection, as sepsis and biliary atresia were ruled out. However, a limitation of this case is the absence of tests for alpha-1 antitrypsin deficiency due to a lack of necessary diagnostic equipment in the country.

Neonatal imaging abnormalities (transfontanellar ultrasound and brain scan) exist in 70% of symptomatic newborns [4]. Intracerebral calcifications are the most common [5]. The primary noninvasive fetal evaluation tool following confirmed or suspected primary maternal CMV infection is fetal ultrasound [1]. This tool, available in most developing countries, is useful for routine structural assessment of the fetus at 18 and 20 weeks of gestation [1]. Ultrasound findings in fetuses with CMV infection include cerebral anomalies, such as cerebral ventriculomegaly, cerebral calcifications, microcephaly, and occipital horn anomalies, as well as non-cerebral anomalies like echogenic intestine, intrauterine growth restriction (IUGR), hepatomegaly, ascites, and cardiomegaly [1].

In this case, a prenatal obstetric ultrasound reported ventriculomegaly. Due to the lack of universal CMV screening, expectant management was followed. Postnatal confirmation was achieved through transfontanellar ultrasound and brain CT, revealing bilateral colpocephaly, periventricular calcifications, and microcephaly. Prenatal findings allow clinicians to anticipate and prepare for a thorough evaluation of the baby once born, performing necessary diagnostic studies to confirm congenital CMV infection. Notably, up to 50% of CMV-infected patients may lack any ultrasound findings, further complicating timely diagnosis [7].

CMV infection during pregnancy can be primary or secondary, with vertical transmission rates of 30% and 0.2% to 8%, respectively [8]. Infections among pregnant women are diagnosed serologically via maternal seroconversion based on the detection of IgG antibodies against CMV. The combination of anti-CMV IgM antibodies and low-avidity anti-CMV IgG antibodies, along with maternal or fetal symptoms, is used to diagnose primary maternal infection. Anti-CMV IgM antibodies are detectable in 70% of CMV-infected newborns. However, CMV polymerase chain reaction (CMV-PCR) provides a more sensitive identification method in saliva, urine, and blood [1].

We consider this case a primary congenital CMV infection, characterized by severe neurological alterations and high viral load in the brain, correlating with greater cytotoxic effects rather than immune response [9]. The negative IgM result in the neonate likely indicates primary congenital infection during the first trimester, allowing IgM levels to become negative and IgG levels to increase as part of the adaptive response. A significant limitation in this case report is the absence of a prenatal serological study for maternal IgM and IgG, and the lack of CMV-PCR diagnosis in the blood or urine of the affected baby due to the unavailability of this technique in the country. However, the clinical and paraclinical findings suggest a severe symptomatic primary CMV infection.

## Conclusions

CMV is a highly heterogeneous congenital infection, posing numerous challenges in prevention, diagnosis, and treatment. This is especially true in a resource-limited healthcare setting. Despite its significance, it has yet to receive the necessary attention, and there is no international consensus for universal screening to prevent future neurological sequelae in symptomatic and asymptomatic patients, who also face a considerable risk of hearing impairment. Keeping CMV on the diagnostic radar for neonatal patients is crucial due to its ability to mimic various clinical entities. In the absence of a vaccine or effective treatment for preventing vertical transmission, preventative measures must be strengthened for all pregnant women with direct contact with children's body fluids.

## References

[1] Naing ZW, Scott GM, Shand A (2016). Congenital cytomegalovirus infection in pregnancy: a review of prevalence, clinical features, diagnosis and prevention. Aust N Z J Obstet Gynaecol.

[2] Stoykova Z, Ivanova L, Cvetkova S, Yordanova D (2020). Congenital cytomegalovirus infection - lessons from a clinical case. Folia Med (Plovdiv).

[3] Kim Y, Kim YM, Kim DR (2023). The multifaceted clinical characteristics of congenital cytomegalovirus infection: from pregnancy to long-term outcomes. J Korean Med Sci.

[4] El Hasbaoui B, Bousselamti A, Redouani MA, Barkat A (2017). Severe neonatal cytomegalovirus infection: about a case. Pan Afr Med J.

[5] Ko HM, Kim KS, Park JW (2000). Congenital cytomegalovirus infection: three autopsy case reports. J Korean Med Sci.

[6] Abolurin OO, Senbanjo IO, Adekoya AO, Ajibola ED (2020). Congenital cytomegalovirus infection as an important cause of infantile cholestatic jaundice: a case report. Pan Afr Med J.

[7] Rodriguez AK, Tjiattas-Saleski L (2023). A case report on congenital cytomegalovirus. Cureus.

[8] Carvalho AA, Silva CB, Martins ML, Santos GC (2021). Congenital cytomegalovirus infection in twin pregnancy. BMJ Case Rep.

[9] Gabrielli L, Bonasoni MP, Santini D (2012). Congenital cytomegalovirus infection: patterns of fetal brain damage. Clin Microbiol Infect.

